# Evaluating the User Preference and Level of Insulin Self-Administration Adherence in Young Patients With Type 1 Diabetes: Experience With Two Insulin Pen Needle Lengths

**DOI:** 10.7759/cureus.8673

**Published:** 2020-06-17

**Authors:** Ayman A Al Hayek, Mohamed Al Dawish

**Affiliations:** 1 Department of Endocrinology and Diabetes, Diabetes Treatment Center, Prince Sultan Military Medical City, Riyadh, SAU

**Keywords:** adherence, diabetes mellitus, insulin injection, needle length, pain

## Abstract

Background

Selecting the appropriate insulin pen needle is important to reduce pain and injection-related adverse events like insulin leakage. It also helps to improve medication adherence and glycemic control in patients with type 1 diabetes mellitus (T1DM).

Objective

This study aimed to compare the 6-mm and 8-mm 32.5-gauge insulin pen needles in terms of glycemic control, pain score, user preference, medication adherence, and injection adverse events in patients with T1DM.

Methods

We conducted a prospective cohort study of 62 patients with T1DM. All patients constituted an experimental group initially and then changed the length of the needle to be part of a self-control group. The glycemic control, visual analog scale (VAS) pain score, Morisky Medication Adherence Scale (MMAS) score, needle attribute score, and injection-related adverse events were measured for all patients with both lengths of needles. Patients were assessed at the baseline visit and followed up for three months. Statistical comparisons were done by the chi-squared test, paired t-test, and paired Wilcoxon test when appropriate with a two-tailed alpha level below 0.05 indicating statistical significance.

Results

With the NanoPass® 32.5-gauge, 6-mm needle (Terumo Corp., Tokyo, Japan), patients had significantly lower glycated hemoglobin (HbA1c) compared to 8-mm needles (7.9% vs. 8.3%; p<0.001). The proportions of patients who reported no hypoglycemic episodes were 22/62 and 9/62, with the 6-mm and 8-mm needles, respectively. The 6-mm needles were better in terms of the following parameters compared to 8-mm needles: mean needle attribute scores (36.7 vs. 24.2; p<0.001), median VAS pain scores (20 vs. 55; p<0.001), insulin leakage (6/62 vs. 20/62; p=0.002), and the MMAS score (4.9 vs. 3.4; p<0.001).

Conclusion

This study provided an overview of the safety, adherence, pain score, and glycemic control relating to the 6-mm and 8-mm insulin needle lengths. Insulin injections using the NanoPass 32.5-gauge, 6-mm needle were associated with lower pain score, higher patient adherence, fewer adverse events, and better glycemic control compared to the 8-mm needle. Therefore, we recommend the use of the NanoPass 6-mm needle for patients with T1DM. Further studies are needed to confirm these findings in patients with type 2 diabetes mellitus (T2DM).

## Introduction

According to the International Diabetes Federation report of 2019, diabetes mellitus (DM) affected about 463 million adult individuals worldwide [1]. About 10% of DM patients suffer from type 1 DM (T1DM), which results from the autoimmune destruction of beta cells of islets of Langerhans [2]. These patients usually present with the disease early in their life, and they require lifelong treatment with intensive insulin therapy to compensate for the shortage in endogenous insulin secretion.

Real-world data from clinical settings have shown that a substantial proportion of diabetic patients fails to achieve optimal glycemic control or the target metabolic control despite taking their medications [3-7]. Poor insulin injection technique is regarded as one of the main reasons for the suboptimal response to insulin therapy for two reasons [8-10]. First, poor injection technique might increase pain perception, thereby reducing patient adherence to medication. Fear of injection has been linked to low adherence to insulin therapy [11,12]. Therefore, selecting the appropriate needle type is important to reduce pain and fear and increase medication adherence as well as glycemic control [13]. Secondly, poor insulin injection techniques might lead to insulin leakage and suboptimal delivery of the insulin dose, and this might affect the level of glycemic control. Hence, researchers have been trying to improve insulin injection techniques for years [14].

Several insulin pen needle types have been developed recently with the aims of easing the insertion into the skin, maximizing insulin delivery to the subcutaneous tissue, reducing the perceived pain, and decreasing injection-related adverse events [13]. Needle manufacturers attempt to reduce needle diameter and length in order to reduce the pain associated with the injection. However, these efforts are often confronted with the challenges of maintaining a suitable inner lumen for insulin passage and delivery without leakage.

Data from the literature suggest that different types of needles, with different diameters, lengths, and geometry, might affect patient preference, adherence to medication, pain perception, and glycemic control [13,15-20]. Despite the advances in needle technology, there has been some hesitance among diabetes healthcare professionals and patients alike regarding the use of shorter needles. We conducted this pragmatic, self-controlled clinical trial to compare the 6-mm and 8-mm insulin pen needles in terms of glycemic control, pain score, user preference, medication adherence, and injection-related adverse events in patients with T1DM.

## Materials and methods

This study has been registered at Prince Sultan Military Medical City, Riyadh the Kingdom of Saudi Arabia. The study was approved by the ethics committee with IRB approval number 1279. All participants provided written informed consent before participating in this study.

Study design, setting, and duration

We conducted a prospective cohort study of patients with T1DM at the Department of Endocrinology and Diabetes, Diabetes Treatment Centre, Prince Sultan Military Medical City, Riyadh. T1DM patients treated at our center during the period from June 2019 to January 2020 were eligible for participation in this study.

Eligibility criteria of the study population

Eligible participants were selected according to the following criteria: (1) patients with a confirmed diagnosis of T1DM, (2) patients aged 14-19 and treated with multiple insulin doses using basal-bolus therapy for at least one year, (3) patients who had never experienced injection of insulin using a needle shorter than 8 mm before, and (4) patients who were able to self-inject their insulin with no physical barriers against the self-injection of insulin. We excluded diabetic patients who did not require long-term insulin therapy and those with physical barriers against self-injection of insulin, including those with loss of visual activity, less handling power, or tremor of the finger.

Outcome measures

The outcome measures of this study were as follows: 

(1) Glycemic Control (HbA1c levels)

Glycated hemoglobin (HbA1c) is a measure of glycemic control in diabetic patients. In our center, HbA1c is measured using the COBAS INTEGRA 400 plus/800 analyzers (Roche Diagnostics, Basel, Switzerland). We obtained the relevant data from the hospital records.

(2) Frequency of Hypoglycemic Events Per Month

Confirmed hypoglycemic episodes were defined as having a blood glucose value of ≤70 mg/dL. Hypoglycemia frequencies were collected using the blood glucose meter software prior to the commencement of the study, and three months after baseline.

(3) 8-mm and 6-mm Needles Attribute Scores

The needle attribute score is a clinical score assessing the overall patient satisfaction about the insulin injection process [21]. Higher scores indicate more satisfaction. The needle attribute scores are based on the following factors: injection comfort, needle quality, ease of inserting a needle into the skin, ease of putting a needle on the pen, least pain when inserting a needle into the skin, least pain when delivering insulin, needle gauge, needle length, and overall satisfaction.

(4) Frequency of Injection-Related Adverse Events

This includes parameters such as insulin leakage, bending of the needle, needle break during injection, bleeding at the injection site, and dribbling from the needle tip.

*(5)* *Morisky Medication Adherence Scale (MMAS)*

The MMAS is an eight-item questionnaire that evaluates patient adherence to the medication. A higher score indicates higher patient adherence to the medications [22].

*(6)* *The 100-mm Visual Analog Scale (VAS)*

The VAS score is an ordinal outcome measure of patient-reported pain score with the value of 100 representing the maximum pain and 0 representing no pain [23].

Study procedure and injection needles

All patients in our center initially used the NanoPass 32.5-gauge, 8-mm needles (Terumo Corp., Tokyo, Japan). At baseline, all patients were examined and assessed for the study outcome measures; then all patients shifted to the 6-mm needles of the same gauge and were followed up for three months. At the end of the follow-up, all patients were assessed for the same outcome measures. Patients did not receive any additional counseling on the injection technique during the study.

Data source

Data were sourced from electronic medical records. HbA1c confirmed hypoglycemia episodes, and the frequency of blood glucose testing was collected in a similar manner at the three-month follow-up visit. Patients self-reported the needle attribute scores, insulin injection-related adverse events, the MAMS score, and the VAS score at baseline and after three months for the 8-mm and 6-mm needles, respectively.

Statistical analysis

Data normality was tested by the Kolmogorov-Smirnov test. For continuous variables, mean and standard deviations were used if normally distributed, or median and interquartile ranges (IQR) if not normally distributed. Categorical variables were expressed as counts and percentages. The comparison between the two needles attribute scores, adverse events score, and the MAMS scores were made using the paired t-test or the Mann-Whitney test according to data normality. An alpha level below 0.05 was used to indicate statistical significance. All analyses were done by the SPSS Statistics software version 25 for Windows (IBM, Armonk, NY).

## Results

Characteristics of the study population

Our study included 62 patients with T1DM for an average of 5.1 years with a mean age of 15.4 years and a mean weight of 58.6 kg. The demographic characteristics of the study population are shown in Table 1.

**Table 1:** Baseline characteristics of the study population Continuous variables are expressed as mean and standard deviation while categorical variables are expressed as counts and percentages SD: standard deviation; DM: diabetes mellitus

Study population variables	Descriptive statistics
Age, year, mean (SD)	15.4 (1.29)
Male gender, n (%)	33 (53.23)
DM duration, year, mean (SD)	5.1 (2.25)
Weight, kg, mean (SD)	58.6 (8.4)
Height, m, mean (SD)	153.6 (6.5)
Body mass index, kg/m^2^, mean (SD)	24.7 (2.5)

Clinical characteristics with the two lengths of needles

Patients had significantly lower HbA1c and fewer hypoglycemic episodes with the NanoPass 6-mm needles compared to the 8-mm needles (Table 2). The common injection sites for 6-mm needles were the abdomen or rotating injections in variable sites, while for the 8-mm needle, the most frequent sites of injections were arms and thighs (Table 2).

**Table 2:** Comparison of both needle lengths in terms of total insulin dose, HbA1c, hypoglycemic episodes, and site of injection Continuous variables are expressed as mean and standard deviation while categorical variables are expressed as counts and percentages SD: standard deviation; HbA1c: glycated hemoglobin

Variables			NanoPass (32.5 gauge, 6 mm)	NanoPass (32.5 gauge, 8 mm)	P-value
Total daily insulin dose, mean (SD)			0.81 (0.16)	0.86 (0.18)	<0.001
HbA1c, %, mean (SD)			7.9 (0.67)	8.3 (0.95)	<0.001
Hypoglycemic episodes per month		0, n (%)	22 (35.5%)	9 (14.5%)	<0.001
		1, n (%)	27 (43.5%)	32 (51.6%)	
		2, n (%)	13 (21%)	18 (29%)	
		3, n (%)	0 (0%)	3 (4.8%)	
Injection site		Abdomen, n (%)	20 (32.3%)	11 (17.7%)	<0.001
		Thigh, n (%)	3 (4.8%)	23 (37.1%)	
		Arm, n (%)	2 (3.2%)	23 (37.1%)	
		Buttocks, n (%)	1 (1.6%)	1 (1.6%)	
		Rotating (more than one), n (%)	36 (58.1%)	5 (8.1%)	

Needle attribute and MMAS score with the two lengths of needles

In terms of the needle attribute score and its individual items, NanoPass 6-mm needle achieved significantly higher scores compared to the 8-mm needle (<0.001; Table 3). Similarly, the MMAS score was significantly higher with the NanoPass 6-mm needle compared to the 8-mm needle (Table 3).

**Table 3:** The needle attribute score and MMAS score with the two lengths of needles SD: standard deviation; MMAS: Morisky Medication Adherence Scale

Scoring method		NanoPass (32.5 gauge, 6 mm)	NanoPass (32.5 gauge, 8 mm)	P-value
Needle attribute score, mean (SD)	Q1: Injection comfort	4.13 (0.61)	2.18 (0.66)	<0.001
	Q2: Needle quality	4.35 (0.54)	2.69 (0.66)	<0.001
	Q3: Ease of inserting needle into skin	3.73 (0.61)	3.03 (0.70)	<0.001
	Q4: Ease of putting needle on the pen	3.74 (0.65)	3.29 (0.61)	<0.001
	Q5: Least pain when inserting needle into the skin	4.39 (0.66)	2.69 (1.18)	<0.001
	Q6: Least pain when delivering insulin	3.92 (0.68)	2.68 (0.98)	<0.001
	Q7: Needle gauge	4 (0.627)	3.15 (0.81)	<0.001
	Q8: Needle length	4.23 (0.58)	2.02 (0.74)	<0.001
	Q9: Overall satisfaction	4.26 (0.63)	2.44 (0.74)	<0.001
	The sum needle attribute score	36.7 (2.44)	24.2 (3.2)	<0.001
MMAS score, mean (SD)	Q1: Do you sometimes forget to take your [health concern] pills?	0.40 (0.49)	0.19 (0.39)	0.011
	Q2: People sometimes miss taking their medications for reasons other than forgetting. Thinking over the past two weeks, were there any days when you did not take your [health concern] medicine?	0.42 (0.49)	0.13 (0.34)	<0.001
	Q3: Have you ever cut back or stopped taking your medication without telling your doctor, because you felt worse when you took it?	0.32 (0.47)	0.16 (0.37)	0.036
	Q4: When you travel or leave home, do you sometimes forget to bring along your [health concern] medication?	0.39 (0.49)	0.42 (0.49)	0.717
	Q5: Did you take your [health concern] medicine yesterday?	0.94 (0.25)	0.98 (0.12)	0.174
	Q6: When you feel like your [health concern] is under control, do you sometimes stop taking your medicine?	0.37 (0.49)	0.32 (0.47)	0.575
	Q7: Taking medication every day is a real inconvenience for some people. Do you ever feel hassled about sticking to your blood pressure treatment plan?	0.48 (0.50)	0.31 (0.46)	0.044
	Q8: How often do you have difficulty remembering to take all your medications?	1.55 (0.84)	0.87 (0.74)	<0.001
	The sum MMAS score	4.9 (2.1)	3.4 (1.5)	<0.001

VAS pain scale with the two lengths of needles

**Figure 1:**
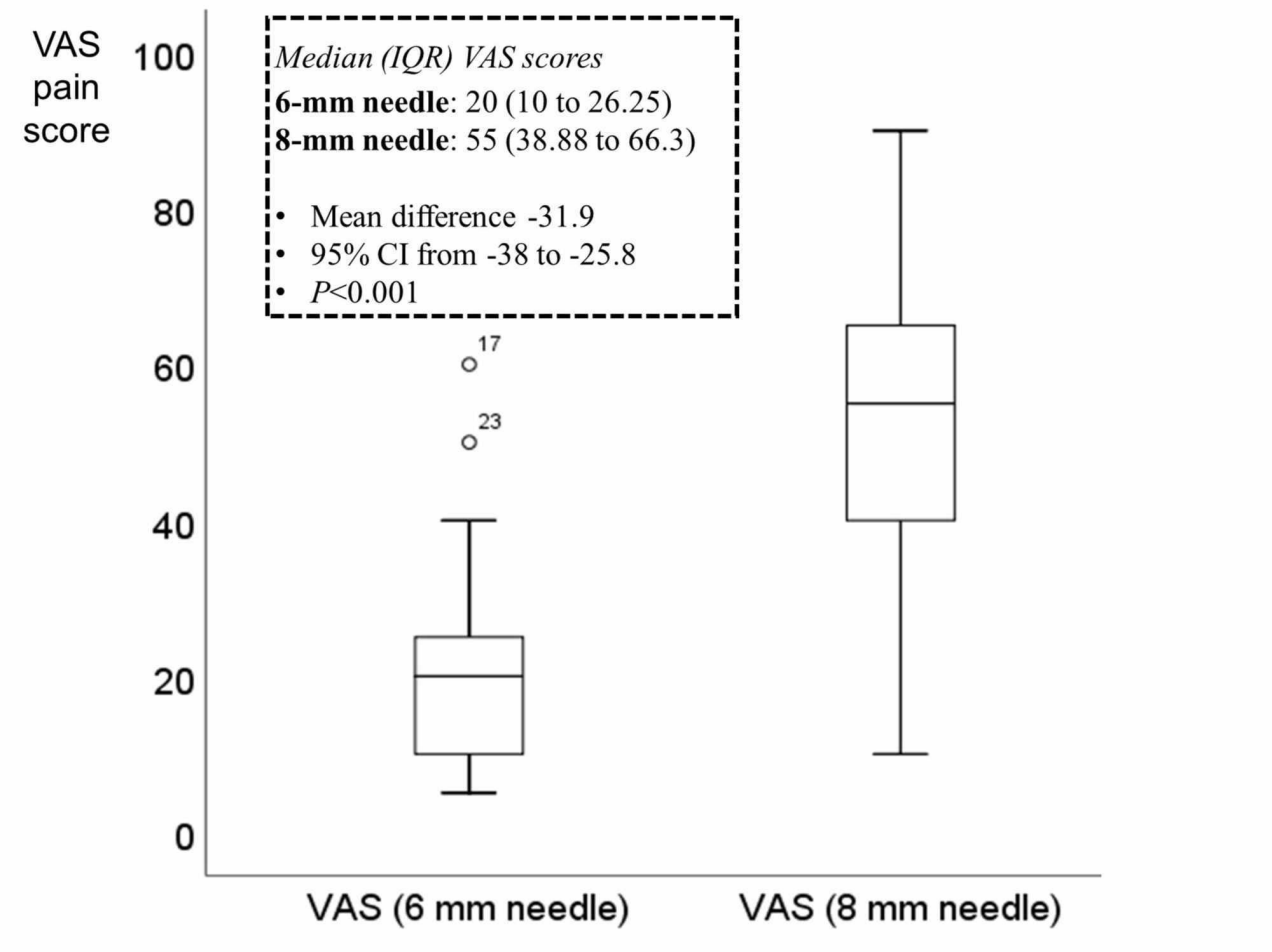
Box plot of the median (IQR) of the VAS score with the two lengths of needles IQR: interquartile range; VAS: visual analog scale; CI: confidence interval

The mean reported VAS pain scores were 52.6 for the 8-mm needle and 20.7 for the 6-mm needle with a mean difference of -31.9 between the two needles, suggesting that NanoPass 32.5 gauge, 6-mm needle is less painful than the 8-mm needle of the same gauge (Figure 1).

Injection-related adverse events with the two lengths of needles

Overall, more frequent injection-related adverse events occurred with the 8-mm needle than the 6-mm needle (Table 4). Insulin leakage was significantly more prevalent in the 8-mm needle (20/62) compared to the 6-mm needle (6/62); this difference was statistically significant (p=0.002).

**Table 4:** Injection-related adverse events with the two lengths of needles

Adverse events	NanoPass (32.5 gauge, 6 mm)		NanoPass (32.5 gauge, 8 mm)		P-value
	Yes	No	Yes	No	
Insulin leakage	6/62	56/62	20/62	42/62	0.002
Bending of the needle	0/62	62/62	3/62	59/62	0.80
Needle break during injection	0/62	62/62	0/62	62/62	NA
Bleeding at the injection site	7/62	55/62	13/49	36/49	0.143
Dribbling from the needle tip	8/62	54/62	15/62	47/62	0.106

## Discussion

Summary of the study findings

This prospective cohort study showed that the NanoPass 32.5 gauge, 6-mm needle is better than the 8-mm needle with the same gauge. The 6-mm needle was associated with better glycemic control, as demonstrated by the better HbA1c levels (7.9% vs. 8.3%). The proportion of patients who reported no hypoglycemic episodes was 22/62 and 9/62 with the 6-mm and 8-mm needles, respectively. No patients reported three hypoglycemic episodes per month with the 6-mm needles, while three patients reported three hypoglycemic episodes per month with the 8-mm needles.

Needle attribute score and VAS pain scores were more favorable with the NanoPass 6-mm needle compared to the 8-mm needle. In terms of the injection-related adverse events, insulin leakage was significantly less frequent with the NanoPass 6-mm needle. Similarly, the MAMS score was higher with the 6-mm needle compared with the 8-mm needle.

The significantly more frequent insulin leakage could explain the difference in glycemic control achieved by the two needles; insulin leakage reduces the amount and deliverability of the injected insulin dose. It has not escaped our notice that the present study showed that shorter needles were not associated with more insulin leakage. This could be explained by the fact that the present study participants were average in terms of weight and BMI. On the contrary, shorter needles might cause insulin leakage in patients who may have a thicker layer of subcutaneous fat. The present study finding should be interpreted cautiously, taking into consideration the average body weight and BMI of the study participants.

The slight improvement in the HbA1c compared to the baseline could be explained by the increased patient adherence to insulin injection as demonstrated by the MMAS score (4.9 vs. 3.5, p<0.001).

Previous studies

Several previous studies have compared different types of needles for insulin injection in DM patients. Since DM is a chronic disease that requires life-long therapy, optimizing the mode of administration of the DM medications is important to achieve less pain and more medication adherence, thereby ensuring better glycemic control and fewer long-term diabetic complications.

Nagai et al. conducted an open-label, randomized, controlled trial of 84 patients with DM [24]. They compared the thin micro tapered needle with the shorter straight needles for insulin injection in DM patients. They found that shorter needles were associated with lower pain scores compared to the thinner micro tapered needles. Although no difference in the glycemic control was found, 60% of the patients preferred the shorter needles, while 19% of the patients preferred the thinner tapered needles, which indicates that needle length is a more important factor than needle diameter. These results are in agreement with our findings that shorter needles (6 mm) are better than a longer needle (8 mm) in terms of the patient-reported VAS pain score.

Hirose et al. conducted a three-way randomized, crossover clinical trial involving 12 healthy Japanese adult males to test whether the use of the 4-mm needles would alter the pharmacokinetics of insulin or C-peptide secretion in non-diabetic adult males [20]. They found that the use of 4-mm needles is unlikely to affect the pharmacokinetic properties of insulin injected by the subcutaneous route.

Kreugel et al. conducted a randomized trial to evaluate the impact of two insulin pen needles on glycemic control and patient preference in 130 insulin-treated type 1 and type 2 DM patients [19]. They found that the shorter needles (5 mm) resulted in less bleeding while the longer needles (8 mm) was associated with less insulin leakage (p=0.01). No clinically significant differences were reported in terms of glycemic control (HbA1c difference: 7.47% vs. 7.59%, respectively).

In another single-blinded study, Præstmark et al. evaluated the impact of needle diameter, length, and needle grinds on the ease of insertion, pain, and blood perfusion. They reported that the shape and design of the insulin needles significantly affected the ease of penetration, pain, bleeding, and skin trauma [16].

Miyakoshi et al. conducted a single-center, open-label, cross-over clinical trial to compare patient preference, pain, and usability between the Micro Fine Plus 31-gauge, 5-mm needle (Nippon Becton Dickinson Co., Ltd., Tokyo, Japan) versus the micro tapered NanoPass 33-gauge, 5-mm needle [17]. They reported no significant differences between the two needle types in terms of patient satisfaction scores. However, the NanoPass needles were superior with less pain, less bruising, less bleeding, less frightening use, and less dribbling of the injected insulin.

Points of strength

The strong points of our study are as follows: (1) we compared the two types of needles in terms of multiple outcomes including the VAS pain score, the needle attribute score, MMAS score, insulin dose, glycemic control, and hypoglycemic events, and (2) the study was self-controlled, i.e., all patients acted as experimental and control participants. This allowed for paired comparisons between the study outcomes. Nonetheless, our study was limited as we focused only on two types of insulin needles, while many other types of needles are available in the market. This should be examined in future studies.

Generalizability and current knowledge

We believe this study contributes to the literature by providing evidence that the NanoPass 32.5 gauge, 6-mm needle might be superior to the 8-mm needles in terms of glycemic control, hypoglycemic events, pain perception, insulin leakage, and patient adherence to the medications. The findings of this study should be interpreted in the context of our strict eligibility criteria; we included patients with T1DM only. Therefore, these results do not apply to patients with type 2 diabetes mellitus (T2DM) who require insulin injection as those patients tend to have different demographic, clinical, and physical characteristics from those with T1DM.

## Conclusions

This study provided an overview of the safety, adherence, pain score, and glycemic control achieved by insulin needles that are 6 mm and 8 mm in length. Insulin injections using the 6-mm needles were associated with lower pain score, higher patient adherence, fewer adverse events, and better glycemic control compared to the 8-mm needles. Therefore, we recommend the use of the NanoPass 6-mm needle in patients with T1DM. Further studies are needed to confirm if our findings are applicable to patients with T2DM.
